# Is Pertussis Infection Neglected in China? Evidence from a Seroepidemiology Survey in Zhejiang, an Eastern Province of China

**DOI:** 10.1371/journal.pone.0155965

**Published:** 2016-05-25

**Authors:** Hanqing He, Pingping Yao, Yang Zhou, Xuan Deng, Jinren Pan

**Affiliations:** 1 Department of Immunization Program, Zhejiang Provincial Center for Disease Control and Prevention, Hangzhou, Zhejiang, China; 2 Department of Microbiology Lab, Zhejiang Provincial Center for Disease Control and Prevention, Hangzhou, Zhejiang, China; Universidad Nacional de la Plata, ARGENTINA

## Abstract

**Background:**

The resurgence of pertussis has occurred in many countries. However, the epidemiological profiles of pertussis cannot be well understood by the current surveillance system in China. This study was designed to investigate the age specific serologic evidence of antibodies against pertussis, and to offer information regarding the existence of pertussis infection in Zhejiang Province, China.

**Methods:**

A cross-sectional serosurvey was carried out in 6 counties of Zhejiang Province during September and October of 2014. The immunoglobulin G-pertussis toxin (IgG-PT) levels were measured quantitatively with a commercially available enzyme-linked immunosorbent assay (ELISA). The antibody activities were expressed in the Food and Drug Administration (FDA)-U/ml and a level ≥30 FDA-U/ml was considered seropositive. An IgG-PT >80 FDA-U/ml indicated recent pertussis infection if the patient had not received immunization with the pertussis vaccine within the last year.

**Results:**

The mean IgG-PT seropositivity rate among the 2107 subjects was 33.32% with a geometric mean concentration of 17.73 (95% confidence interval: 16.90–18.60) FDA-U/ml. The difference in the seropositivity rates reached significant means among the different age groups (waldχ^2^ = 198.41, *P*<0.0005), and children aged 3 years had the highest percentage (63.24%) of undetectable IgG-PT level. Of the 1707 subjects ≥3 years of age, 169 (9.90%) had evidence of a recent infection. The highest proportion of IgG-PT levels ≥80 FDA-U/ml was found in ≥60 years age group followed by 11–15 and 16–25 years age groups.

**Conclusions:**

This study indicates the rather lower IgG-PT level sustained 1 year after the acellular pertussis vaccine booster dose, and substantial proportion of population susceptibility to pertussis in Zhejiang Province, China. Moreover, pertussis infection is not uncommon; it was estimated that 10% of subjects were recently infected approximately within the last 100 days. We highly suggest that the surveillance capacity should be strengthened and consider introducing booster dose that protect against pertussis in 6 years old children.

## Introduction

Pertussis, caused by the gram-negative bacterium *Bordetella pertussis*, is a common infectious diseases worldwide [1–3]. In China, pertussis is a notifiable disease that mainly affects infants and younger children. Like many other places in the world, pertussis was very common 50 years ago in Zhejiang Province, an eastern coastal province of China. About 40,000 pertussis cases were reported, with 86 of those cases resulting in death each year before the year 1978.

The incidence of pertussis decreased rapidly after the delivery of the whole cell pertussis vaccine combined with diphtheria and tetanus toxoids (DTwP) that was introduced in the Expanded Program on Immunization (EPI) in China since 1978. The annual reported pertussis incidence rate had decreased from 143.11 per 100,000 individuals (1954–1977) to 33.92 per 100,000 individuals (1978–1993) with less than 1 case per 100,000 individuals reported since 1994 in Zhejiang Province [4]. In 2007, a combined diphtheria-tetanus-acellular pertussis vaccine (DTaP) was introduced in EPI of China [5]. Both DTaP and DTwP were used in Zhejiang Province during 2007–2009, and only DTaP was administered after 2010. DTaP was now administered in the 3^rd^, 4^th^, and 5^th^ months of infancy, and a booster dose was given between 18 and 24 months. Since 1995, the reported coverage rates of the pertussis vaccine were higher than 95% in Zhejiang Province. Meanwhile, the incidences of pertussis had remained very low (0.08–0.89 per 100,000 individuals from 1995 to 2013), where most cases occurred in individuals aged <1 year old.

However, the resurgence of pertussis has occurred in many other countries, including some developed countries, despite a high coverage with DTaP or DTwP [6–8].

More importantly, the number of adolescent and adult cases has recently accounted for an increased proportion of the total cases, compared with previous periods [1, 6]. The improvement in diagnostics, pathogen adaptation, and waning immunity after vaccination may be the main reasons for this increase [9]. Previous studies have indicated that the incidence of pertussis infection may have been underestimated in China, particularly in adolescents and adults with atypical symptoms [10–12].

Previously, most pertussis cases were clinically diagnosed and laboratory methods were not routinely used in China. Therefore, the true epidemiological profiles of pertussis cannot be well understood by the current surveillance system in China [13]. The low incidence of pertussis reported may also not reflect the real disease burden in Zhejiang Province.

Serosurveys are widely used to estimate the incidence rate of pertussis as they can capture both symptomatic and asymptomatic infection [9, 14, 15]. Pertussis toxin (PT) is the most specific antigen for pertussis and cross-reacting antigens have not been reported, therefore the immunoglobulin G (IgG) of PT will likely provide a true indication of pertussis prevalence. Several seroprevalence studies for pertussis have been undertaken in China; however, the age-specific prevalence of infection was inconsistent between different areas [9–12]. These differences may due to the heterogeneity of social demography, implement of the immunization program, differences in study methodology and the year of study. Therefore, we cannot simply apply these results to Zhejiang Province to uncover the epidemiological characteristics of pertussis infection.

To our knowledge, a pertussis seroepidemiological study of general population in Zhejiang Province has not been reported. Therefore, the true pertussis epidemiological profile in Zhejiang Province is not clear. This investigation was designed to offer information regarding pertussis infection in Zhejiang Province and to provide further insight on effective immunization strategies.

## Methods

### Study population and design

This cross-sectional serosurvey was carried out in 6 counties of Zhejiang Province from September 2014 to October 2014, after the geographical and economic statuses were considered. An age-stratified sampling method was used with subjects randomly selected from each county based on the following age groups: at birth (umbilical cord blood), 1–2 years, 3 years, 4–5 years, 6–7 years, 8–10 years, 11–15 years, 16–25 years, 26–35 years, 36–59 years, and ≥60 years. Participants were requested to give a blood sample and to fill out a questionnaire, and at least 30 samples were required from each age group in each county according to the estimation of the sample size. Sera were transferred on ice boxes and were stored at -70°C until processed.

The study was approved by the ethics committee of Zhejiang Provincial Center for Disease Control and Prevention and was conducted in accordance with Good Clinical Practice guidelines and Declaration of Helsinki. Written informed consent was obtained from all participants or the guardians (for children ≤18 years) prior to enrollment in the study.

### Laboratory methods

The IgG-PT levels were measured quantitatively by a commercially available enzyme-linked immunosorbent assay (ELISA) test kit (Virion\Serion, Germany) according to the manufacturer’s protocol. The antibody activities were expressed in the U.S.A. Food and Drug Administration (FDA)-U/ml. The IgG-PT results were interpreted as positive or negative according to the manufacturer's guidelines. Subjects bearing >30 FDA-U/ml of IgG-PT were considered seropositive.

### Statistical analysis

A geometric mean concentration (GMC) was calculated using a log transformation and was reported as a back transformed titer. Values below the detection threshold (10 FDA-U/ml) were assigned half of the threshold value (5 FDA-U/ml) in calculation. The binomial logistic regression model with likelihood ratio test was applied to compare seropositivity varies between age groups. Chi-Square test for trend was used to assess the association between the estimated increasing of infection rates with the age growing. One-way analysis of variance was used to test for differences in the GMC levels among the age groups. All statistical tests were two-sided and considered statistically significant at *P* value < 0.05. The cut-off level of 80 FDA-U/ml was applied to indicate the recent infection of pertussis according to previous studies [16, 17]. The estimated incidence of pertussis infection was limited in subjects aged ≥3 years in order to avoid the interference with vaccination-induced or maternally derived antibodies. All data were handled using EpiData (version 3.1) and the statistical analysis was performed by SPSS (Version 18.0, Chicago, USA).

## Results

### Study population

A total of 2118 subjects were enrolled in the study with 11 subjects excluded from the analysis due to poor quality of their blood samples. Of the 2107 subjects included in the following analysis, 985 were male and 1122 were female. Their ages ranged from 0 to 91 years old (median: 11 years old) with at least 180 subjects in each age group. There were 208 subjects who had samples collected from their cord blood, and the number of the subjects aged ≥3 years was 1707. Therefore, the corresponding estimated pertussis infection rate was based on the 1707 subjects.

### Prevalence of IgG-PT

The mean seropositivity rate of IgG-PT among the 2107 subjects was 33.32% with a GMC of 17.73 FDA-U/ml (95% confidence interval [*CI*]: 16.90–18.60 FDA-U/ml). The provincial level rate of seropositivity was estimated as 43.02% (95% *CI*: 40.91%–45.14%) according to the age group proportional distribution in Sixth National Population Census [18]. No significant differences were observed between male and female subjects regarding the seropositivity rate (33.30% vs. 33.33%, *Wald*χ^2^ = 0.00, *P* = 0.987) or GMC levels (18.00 FDA-U/ml vs. 17.49 FDA-U/ml, *t* = 0.58, *P* = 0.564). However, the seropositivity rates and GMC levels for IgG-PT both reached significant means among the different age groups (*Wald*χ^2^ = 198.41, *P*<0.0005; *F* = 33.10, *P*<0.0005). There were 702 subjects (33.74%) with undetectable IgG-PT levels (lower than 10 FDA-U/ml), and children aged 3 years old had the highest percentage of undetectable IgG-PT levels (63.24%).

**Fig 1:**
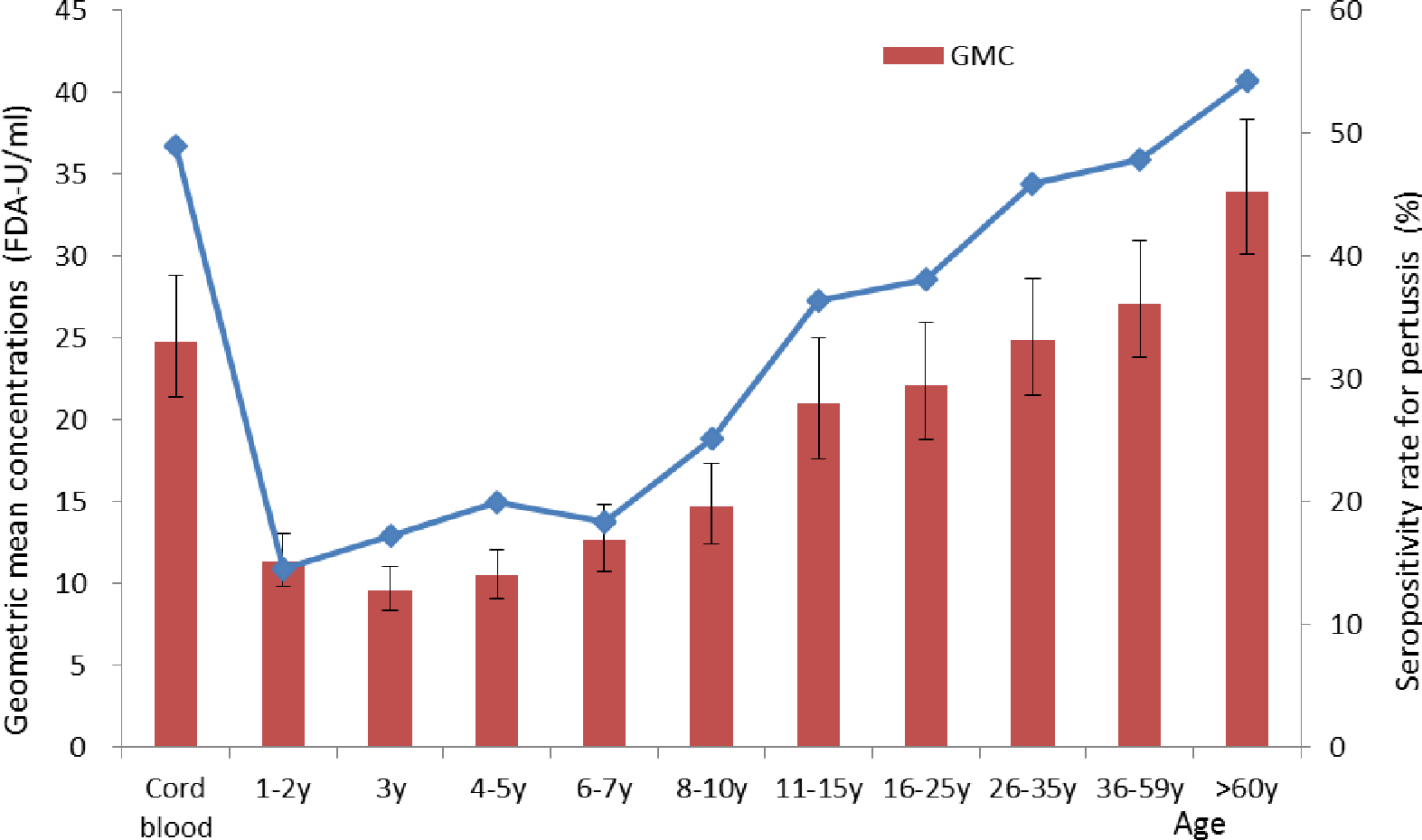
The age-specific seropositivity rates and geometric mean concentrations of antibodies against pertussis in Zhejiang Province, China, 2014.

Fig 1 shows the seropositivity rates and GMC levels for IgG-PT by age group. The rate of infants born with positive IgG-PT antibodies was 49.04%, while the rate decreased rapidly to the lowest level (14.58%) in the 1–2 years age group. The seropositivity rate increased in the age groups above 2 years of age, and a V-curve distribution was found among the different age groups. A low IgG-PT seropositive rate of (9.61%-14.70%) was observed in subjects aged 0–10 years. The age-specific GMC distribution generally followed the trend of seropositivity, with the lowest level in children aged 3 years (9.61 FDA-U/ml).

### Pertussis infection

The estimated infection rates for the 1707 subjects ≥3 years old are shown in Table 1. There were 169 subjects (9.90%) with an IgG-PT level ≥80 FDA-U/ml which implies recent infection [16, 17]. The lowest frequency of antibody level ≥80 FDA-U/ml occurred in subjects of 3 years age (3.78%) where the proportion increased in older age groups. This trend between the estimated infection rates and the increasing age reached significant means (Trend χ^2^ = 24.08, *P*<0.0005).

**Table 1:** The rate of pertussis infection by age group in Zhejiang Province, China, 2014.

Agegroup	No. of subjects	≥80 FDA-U/ml	
		*n*	Percent(%)
**3 y**	185	7	3.78
**4–5 y**	205	8	3.90
**6–7 y**	190	15	7.89
**8–10 y**	195	18	9.23
**11–15 y**	184	26	14.13
**16–25 y**	189	23	12.17
**26–35 y**	181	19	10.50
**36–59 y**	192	22	11.46
**≥60 y**	186	31	16.67
Total	1707	169	9.90

## Discussion

Pertussis remains an important ongoing public health concern worldwide. However, the disease burden has still not been well addressed, especially in developing countries [19]. In China, the reported incidence of pertussis has continued to be very low (<1 per 100,000 individuals) in the past two decades, but the true pertussis disease burden is not clear due to the insensitive surveillance system [9, 11].

In the present study, we demonstrated a 33.32% seropositivity rate for IgG-PT in overall subjects, with the estimated provincial rates of seropositivity was 43.02% (95%*CI*: 40.91%–45.14%). The rate was consistent with studies carried out in Guangdong (32.51%) [20], Shanxi (31.80%) [21] in China, and result amongst the healthcare workers in Spain (31.2%) [22]. While our result was higher in comparison to reports from Wang et al. [9], Zhang et al. [10], and Xu et al. [11], this may be explained by differences in the studied populations, various methods used, etc. PT is the specific maker for pertussis infection or vaccination, the vaccine induced-antibodies are short-lived and wane to lower levels about within 1 year [15]. Our results showed that the IgG-PT antibody levels were relatively lower in younger children than in other age groups, and the highest percentage of undetectable antibody level was found in the 3-year age group (1 year after booster dose). The high proportion of subjects with an absence in detectable antibodies in our study may indicate a possible risk of susceptibility at the population level.

Our study showed an increase in the level of IgG-PT antibodies with increasing age after 3 years old. However, no booster dose was routinely used for the pertussis vaccine after 18–24 months of age in China as yet. The rise may attributable in part to the lower immunity from DTaP vaccine which was introduced in EPI to replaced DTwP vaccine in recent years [23]. The interpersonal contact increased following the age growing, and asymptomatic transmission may also be the explanation for this results [24]. Therefore, the rise trend might be due to an age-related cumulative exposure to pertussis. The high level of IgG-PT antibodies in age groups not targeted for vaccination even can be interpreted as more chance of pertussis natural infection [25]. According to previous studies [15, 16] and the ELISA kit used by our laboratory, we applied the IgG-PT level ≥80 FDA-U/ml as the indication of recent infection with pertussis. We estimated that 9.90% of subjects older than 3 years of age might have a recent infection. The estimated infection rates remain stable (from 14.88% to 8.44%), even we changed the cut-off value from 70 to 100 FDA-U/ml [17]. Higher infection of pertussis was also found in Tianjin, China, according to the active symptom surveillance [12]. This suggests that pertussis is not uncommon in China, and the germs are still circulating in community. The highest estimated incidence of infection was in individuals aged ≥60 years with another peak incidence appearing among those aged 11–15 years. The age distributions for the estimated infection rates were different with that of the reported pertussis incidence (almost all reported cases were aged <1 year old). The similar estimated infection rates of pertussis were reported in Netherlands, and huge gap were also found with the reported incidence rates [17].

In Zhejiang Province, like many other places in China, the notification incidence rates of pertussis were less than 1 per 100,000 population during the period of 2005–2014. The reported rates were thousands of times lower than the estimated infection rates. One explanation for this discrepancy is the antibody responses indicate the presence of both symptomatic and asymptomatic infection, and thus the IgG-PT levels reflect exposure to pertussis rather than the true clinical episode [11]. Another reason for the gap may due to under-reporting of milder cases, especially in adolescents and adults who usually present with atypical symptoms. Cases of pertussis have also been under-reported and under-diagnosed in many other countries [26]. The true incidence of pertussis is most likely underestimated according to the passive diseases surveillance system [20]. Only about 5% of the confirmed pertussis cases were properly diagnosed as pertussis at their first medical visit in China [12]. Taken together, the current surveillance system for pertussis may not allow us to assess the disease burden, therefore, a routine laboratory-based surveillance system should be established. A better understanding of pertussis trends is very important to assess current immunization strategies as well as for implementing future policies.

The increasing pertussis infection rate by age may be in part attributable to the waning immunity for pertussis after immunization [23]. Vaccination protects children against disease but may not help them avoid infection and transmission of pertussis [27]. Asymptomatic transmission is the most important explanation for the resurgence of pertussis, because an aP vaccine which may blocks symptomatic disease but not for asymptomatic transmission [23, 28]. Pertussis is commonly considered as a childhood disease. However, our results show that the estimated recent pertussis infection rate was > 7% in children older than 6 years, and it reached a higher level (>10%) in subjects over 11 years old. The incidence of pertussis infection in adolescents and adults is not given enough attention [29]. All of the reported cases of pertussis in Zhejiang Province were in infants and children <5 years old. Adults infected with pertussis may present with milder symptoms or seek medical attention after coughing for several weeks, making it easy to under-reporting. These adults can become an important reservoir for pertussis transmission from adults to young infants easily through asymptomatic infection [16, 30]. Asymptomatic pertussis infections are also common in adolescents in China, and the asymptomatic transmissions are more prevalent than previously documented in school [10].

Adolescent booster vaccinations, cocooning, or maternal vaccinations are suggested in other countries to further control the circulation of pertussis [31, 32]. The booster doses of tetanus toxoid, reduced diphtheria toxoid, and acellular pertussis (Tdap) were introduced in the United States during 2005, which may help to control the incidence of pertussis infection in adolescents [33]. Our results also show that adolescents, young adults, and older individuals have a higher recent infection rates compared to the overall subjects. In consideration of adolescents and young adults have become new high-risk populations for pertussis in China [12], future work should focus on reinforcing the immunization program, especially among adolescents.

Based on our findings of the lower IgG-PT level after immunization and the rising estimated infection rates in individuals >6 years age, routine booster doses against pertussis should be considered in our EPI program. This can be easily implemented to replace the current reduced diphtheria toxoid and tetanus toxoid (DT) with DTaP at 6 years age in the EPI program in China. In order to further control pertussis transmission, vaccination with Tdap for high-risk adults, such as pregnant women, should also be considered [34].

### Limitations

Our cross-sectional study does not allow us to make a causal inference. Thus, a prospective study should be conducted to further understand the epidemiological characteristics of pertussis. Our analysis was not carried out by each individual immunization status, which may further limit the understanding of immunity following vaccination. However, the child immunization coverage has generally been >95% in Zhejiang during the last 10 years. Therefore, we believe that this did not prevent us from drawing an appropriate conclusion. In order to address the risk of pertussis infection, further study is still needed to explore the correlation between IgG-PT levels and real protection.

## Conclusions

This study indicates the rather lower IgG-PT level sustained 1 year after the aP vaccine booster dose and the substantial proportion of population susceptibility to pertussis in Zhejiang Province, China. It is demonstrated that approximately 10% of subjects were recently infected with pertussis while the disease burden may be underestimated remarkably. Pertussis infection is not uncommon, particularly in adolescents and older adults. We highly recommend establishment of a routine laboratory-based surveillance system that monitors the pertussis burden, and consider introducing booster dose that protect against pertussis in 6 years old children.

## Supporting Information

S1 Data Data set for a cross-sectional serosurvey of pertussis in Zhejiang Province 2014. (SAV)
